# Neurobrucellosis with transient ischemic attack, vasculopathic changes, intracerebral granulomas and basal ganglia infarction: a case report

**DOI:** 10.1186/1752-1947-4-340

**Published:** 2010-10-25

**Authors:** Asuman Sengoz Inan, Nurgul Ceran, Ilknur Erdem, Derya O Engin, Seniha Senbayrak, Seyfi C Ozyurek, Pasa Goktas

**Affiliations:** 1 Department of Infectious Diseases and Clinical Microbiology, Haydarpasa Numune Training and Research Hospital, Tıbbiye Street, 34668, Istanbul, Turkey; 2 Department of Infectious Diseases and Clinical Microbiology, Namık Kemal University, Namik Kemal Street, 59100 Tekirdag, Turkey

## Abstract

**Introduction:**

Central nervous system involvement is a rare but serious manifestation of brucellosis. We present an unusual case of neurobrucellosis with transient ischemic attack, intracerebral vasculopathy granulomas, seizures, and paralysis of sixth and seventh cranial nerves.

**Case presentation:**

A 17-year-old Caucasian man presented with nausea and vomiting, headache, double vision and he gave a history of weakness in the left arm, speech disturbance and imbalance. Physical examination revealed fever, doubtful neck stiffness and left abducens nerve paralysis. An analysis of his cerebrospinal fluid showed a pleocytosis (lymphocytes, 90%), high protein and low glucose levels. He developed generalized tonic-clonic seizures, facial paralysis and left hemiparesis. Cranial magnetic resonance imaging demonstrated intracerebral vasculitis, basal ganglia infarction and granulomas, mimicking the central nervous system involvement of tuberculosis. On the 31st day of his admission, neurobrucellosis was diagnosed with immunoglobulin M and immunoglobulin G positivity by standard tube agglutination test and enzyme-linked immunosorbent assay in both serum and cerebrospinal fluid samples (the tests had been negative until that day). He was treated successfully with trimethoprim and sulfamethoxazole, doxycyline and rifampicin for six months.

**Conclusions:**

Our patient illustrates the importance of suspecting brucellosis as a cause of meningoencephalitis, even if cultures and serological tests are negative at the beginning of the disease. As a result, in patients who have a history of residence or travel to endemic areas, neurobrucellosis should be considered in the differential diagnosis of any neurologic symptoms. If initial tests fail, repetition of these tests at appropriate intervals along with complementary investigations are indicated.

## Introduction

Brucellosis is still a common zoonotic infection in many parts of the world, including North and East Africa, the Middle East, South and Central Asia, South and Central America, and the Mediterranean countries of Europe [1]. Human brucellosis, typically, is acquired by ingestion of unpasteurized milk or cheese, by infected aerosols or through occupational exposure to infected animals; in particular, sheep, goats, swine, camels and cattle. Central nervous system involvement is a rare but serious manifestation of brucellosis. Meningitis (acute, subacute and chronic), meningoencephalitis, brain abscess, epidural abscess, myelopathy, polyradiculitis, mononeuritis and vascular involvements have been reported as main clinical manifestation of neurobrucellosis [2,3]. We present a case with transient ischemic attack (TIA), intracerebral vasculopathy granulomas (which are extremely rare entities, with only a few cases reported in the literature), seizures and cranial nerve paralysis.

## Case presentation

A previously healthy, 17-year-old Caucasian man suffered headache, weakness in his left arm, hypoesthesia, speech disturbance and imbalance, which resolved in a few hours, and he was prescribed an anxiolytic drug by a doctor. Two weeks after this event, he was admitted to our hospital due to worsening headache accompanied by double vision, nausea, vomiting and fever. Physical examination on admission revealed fever (38°C), doubtful neck stiffness and left abducens nerve paralysis. Kerning and Brudzinski signs were negative and all the other systemic findings were non-specific. Laboratory studies showed a leukocyte count of 5.9 × 10^3^/μL (neutrophils 55%, lymphocytes 45% and monocytes 5%), an erythrocyte sedimentation rate of 2 mm/h, and a C-reactive protein of 3.0 mg/dL (normal < 5.0 mg/dL). Blood chemistry including liver enzymes and alkaline phosphatase, prothrombin time, fibrinogen, antithrombin III, protein C, protein S, activated protein C resistance and antiphospholipid antibodies were within the normal ranges. Immunologic markers including immunoglobulins, C3, C4, cryoglobulin, and antinuclear antibodies were negative. The chest X-ray, cranial magnetic resonance imaging (MRI) with contrast, abdominal ultrasound and echocardiography were normal.

A lumbar puncture was performed and the investigation of cerebrospinal fluid (CSF) showed 104 cells/mL (lymphocytes, 90%), a protein concentration of 250 mg/dL, a glucose level of 22 mg/dL and the concurrent blood glucose level of 72 mg/dL. Gram stain and Ziehl-Nielsen stain, culture for bacteria, mycobacteria and fungi were all negative. Serum and CSF Rose-Bengal, standard tube agglutination (STA) test, Coombs' test, enyzyme-linked immunosorbent assay (ELISA) for human immunodeficiency virus 1 and 2, Treponema pallidum hemagglutination assay (TPHA) and Venereal Disease Research Laboratories (VDRL) tests were negative. The purified protein derivative (PPD) skin test was positive (11 × 11 mm). The diagnosis was considered as possible tuberculosis meningitis and our patient was empirically treated with isoniazid 600 mg, rifampicin 600 mg, pyrazinamide 1500 mg, ethambutol 1500 mg and prednisolone 50 mg, daily. The fever subsided after the third day and other clinical symptoms (such as double vision and headache) resolved after six days of therapy.

Although there was no worsening of his general condition, fever reappeared on the tenth day. STA and ELISA *Brucella *IgM and IgG antibodies were negative again and these tests were repeated at weekly intervals both in the samples of serum and CSF. The blood and CSF cultures for bacteria including *Brucella *yielded no growth.

**Figure 1:**
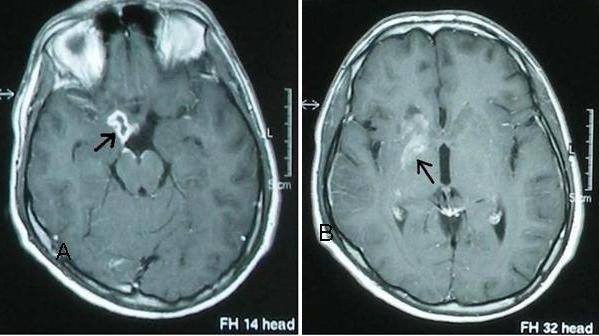
**Post contrast T1-weighted MRI shows (A) a granuloma with peripheral enhancement in the genus of the right capsula interna, and (B) an infarct in the basal ganglion due to vasculitis **.

On day 30 of his admission, our patient developed generalized tonic-clonic seizures. The mild amplitude slow wave activity was detected over the right hemisphere on an electroencephalogram. Cranial computed tomography (CT) showed a 1 cm diameter hypodense nodule at left temporal lobe and a 1 cm hypodense nodule next to the right thalamus anterior internal capsid (the nodules were reported as tuberculomas by staff of the radiology department). The following day he experienced facial paralysis, left hemiparesis and headache. Fever (38.2°C) and neck stiffness were observed. MRI demonstrated an abnormal contrast enhancement in the right sylvian fissure, millimetric nodular lesions in the genus of the right capsula interna on T1-weighted images, hyperintense infarct regions (about 10 mm in right lentiforme nucleus and 20 mm in right caudal nucleus) on T2-fluid attenuated inversion recovery (FLAIR)-weighted images (Figure 1). The lesions were reported as the ischemic process due to vasculitis secondary to tuberculosis and tuberculoma. On the same day a fourth lumbar puncture (LP) was performed and in the evaluation of CSF, the white blood count was 348/mL (lymphocytes 80%), protein 258 mg/dL, glucose 20 mg/dL, and the concurrent blood glucose was 88 mg/dL. Rose-Bengal, STA (1/160 in serum, 1/80 in CSF) and ELISA IgM and IgG antibodies were positive in both serum and CSF samples. Our patient was requestioned for specific antecedents of systemic brucellosis; he was a student from Southeastern Anatolia, where the disease is endemic. A diagnosis of neurobrucellosis was considered and treatment with trimethoprim/sulfamethoxazole (TMP/SMX) 160/800 mg three times a day, doxycyline 200 mg/day and rifampicin 900 mg/day was initiated. Fever disappeared ten days after the therapy. Other signs and symptoms improved completely at the end of the sixth month of therapy. Our patient remains free of any complaints to date (a followup period of three years).

## Discussion

Brucellosis still remains an important health problem in developing countries and its incidence is increasing in Turkey. Approximately 5600 cases annually were reported between 1987 and 1996 (incidence: 9.6/100,000) and around 13,800 cases annually were reported to the Turkish Ministry of Health between 1997 and 2006 (incidence: 20.1/100,000). The seropositivity of brucellosis is 2.6 to 14.4% for the general Turkish population [4].

The frequency with which *Brucella *attacks the CNS is relatively low, with a reported incidence of 1.3% to 13% [2,5]. Of the 101 brucellosis patients followed by our clinic (between 2001 and 2008), eight had CNS involvement (7.9%). Neurobrucellosis may involve several areas of the central and peripheral nervous systems and may develop at any stage of the disease. The most common presentation is a typical meningitis or meningoencephalitis that has an acute and subacute onset, and can occur either as the only site of infection or as part of systemic disease [2,3]. The involvement of one or more cranial nerves, generally the eighth, has been noted in the cases, especially as a manifestation of basal meningeal infection, while involvement of the sixth nerve is very rare [6]. Our patient was admitted to our clinic with an acute meningoencephalitis and sixth nerve palsy and he experienced facial paralysis at the followup period, consistent with his basal involvement.

Cerebrovascular involvement of brucellosis is explained by two mechanisms: the first mechanism is a hemorrhagic stroke caused by rupture of a mycotic aneurysm, which is probably a consequence of embolic phenomenon from brucellar endocarditis; and the second is an inflammatory process of the vessels or venous system resulting in lacunar infarcts, small hemorrhages, or venous thromboses. It was reported that there were only a few cases in the literature of vasculopathic involvement in neurobrucellosis [5,7-9]. Our patient gave a history of weakness in the left arm, hypoesthesia, speech disturbance and imbalance, which disappeared completely in a few hours; so this episode was defined as a TIA. He experienced generalized tonic-clonic seizures, facial paralysis, left hemiparesis during the following period. MRI demonstrated lesions compatible with cerebral vasculitis. Although recently vasculitic involvement in the cerebral arterial system was detected with digital subtraction angiography in one case, angiography findings of our patient showed no abnormality as in the other reported patients in the literature. Al-Deep *et al*. [5] described a case with left hemiplegia and CT demonstrated a frontoparietal infarct, but cerebral angiography was normal. The authors proposed that normal findings of digital subtraction angiography was consistent with vasculitic involvement of deep penetrating cerebral vessels.

Granulomas within the brain parenchyma are extremely uncommon and have been reported in only four cases (Table 1). Ciftci *et al*. [10] described a case with visual impairment, bilateral hearing loss, hyperprolactinemia, meningitis and a sellar and suprasellar mass, who was treated successfully. Sohn *et al*. [11] reported two cases of neurobrucellosis with an intracerebral granuloma due to a marine mammal *Brucella sp*. Another case report about a mass lesion in the parietal lobe due to brucellosis was described by Miguel *et al*. [12]. Al-Sous *et al*. [13] presented a case with neurobrucellosis who had headache, deafness, papilledema, sensory ataxia, areflexia, and a granuloma in the suprasellar region. The authors reported a complete recovery with documented radiologic improvement after four months of medical treatment. On admission MRI was normal, but a second MRI revealed cerebellar granulomas in the patient.

**Table 1:** Cases of neurobrucellosis with intracerebral granuloma.

Author/year	Signs and symptoms	Age/sex	Lesion	Analysis of granuloma, blood, CSF samples	Treatment/duration/result
Ciftci *et al*. [10] 1998	Visual impairment, bilateral hearing loss, hyperprolactinemia, meningitis	25-year-old female	Sellar and suprasellar mass	STA, 1/320, and 1/160 in blood and CSF, respectively 190 cells/mL (lymphocytes), protein 227 mg/dL, glucose 18 mg/dL in CSF	TS, RIF, 10 weeks, hearing loss

Sohn *et al*. [11] 2003	Periorbital pain, headache, generalized tonic-clonic seizure	26-year-old man	3 × 3 cm left frontal lobe mass	Brucella spp. in granuloma culture STA, 1/160, in blood	Surgery + TS, RIF, 2 months, recovery
	
	Headache, nausea, vomiting, progressive visual function deterioration	15-year-old boy	Granulomas in the left occipital and parietal lobes (TI+contrast)	Brucella spp. in granuloma culture Serology:negative	DS,RIF, GM (1 month and then TMP/SMX), 1 year, visual acuity deficits and brain atrophy

Al-Sous *et al*. [13] 2004	Headache, deafness, papilledema, sensory ataxia, areflexia	30-year-old female	Enhancing granuloma suprasellar region(TI+contrast)	STA, 1/1280, 2ME, 1/640, in CSF Protein level: 80 mg/dL in CSF	DS,RIF, TMP/SMX, 4 months, complete recovery

Miguel *et al*. [12] 2006	Weakness of the right upper and lower limbs, headache, Broca's aphasia	39-year-old male	3 cm enhancing mass lesion in the left parietal lobe (T1,T2 + contrast)	IgM and IgG antibodies by ELISA in CSF and blood 10 cells (mostly lymphocytes), protein 76 mg/dL, glucose 55 mg/dL in CSF	DS, RIF, GM, 2 months, right hemiparesis

Present case	Headache, weakness in the left arm, hypoesthesia, speech disturbance, imbalance, fever, diplopia, 6.7.cranial nerves involvement, generalized tonic-clonic seizures	17-year-old boy	A granuloma in the genus of the right capsula interna (T1-contrast)	IgM and IgG antibodies by ELISA i n the CSF and blood STA, 1/160, and 1/80 in blood and CSF, respectively 104 cells (lymphocytes, 86%), protein 250 mg/dL, glucose 22 mg/dlL in CSF	DS, RF, TMP/SMX, 6 months, complete recovery

**Abbreviations **DS: doxycyline; GM: gentamycine; RIF: rifampicin; TMP/SMX: trimethoprim/sulfamethoxazole; TS: tetracycline.

The specific diagnosis of neurobrucellosis is based upon seroagglutination and blood and CSF cultures (positive <15% of the cases), which have relatively low sensitivities. The finding of *Brucella-*specific antibodies in the CSF is highly indicative of neurobrucellosis; however, since the antibodies are sometimes present at low levels, agglutination tests commonly performed in the diagnosis of the disease may give false-negative results. ELISA is the recommended test whenever neurobrucellosis is clinically suspected, even though agglutination tests and cultures were negative [3]. However, in contrast to previously reported cases, IgM and IgG antibodies were negative by ELISA as well as Coombs' test, STA and culture negativity in our patient until the 31st day of the follow up. Unknown host factors, the relatively short duration of his illness, and the applied steroid therapy may be responsible for his lack of serologic response, emphasizing the importance of high index of suspicion of illness and repetition of the diagnostic tests at appropriate intervals, and the use of novel diagnostic technology, and, if possible, a molecular-based method appear to be promising.

Tuberculosis is also an endemic disease in Turkey. Neurotuberculosis includes three clinic categories: meningitis, intracranial granuloma and spinal tuberculous arachnoiditis. The disease presents with a CSF formula characterized by lymphocytic pleocytosis, lowered glucose concentration, and a high protein content, similar to neurobrucellosis. Hemiparesis (40.5%) and cranial nerve palsies (35.7%) were the most common neurological deficits. The prompt diagnosis and antibiotic treatment of tuberculous meningitis save lives [14]. For this reason, careful evaluation for CNS tuberculosis and brucellosis is essential in a patient suspected of any of these probable diagnoses, especially if the empirical therapy fails.

There is no general consensus regarding the best treatment for neurobrucellosis, but the use of doxycycline in combination with two other drugs (rifampicin, TMP/SMX, ceftriaxone) for at least three months is recommended [3]. The mortality rate of neurobrucellosis in the post-antibiotic era is 0 to 5.5%, but permanent neurological deficits, particularly deafness, are common [2]. Our patient was treated with doxycyline, rifampicin and TMP/SMX for six months, without any sequelae.

## Conclusions

Our patient illustrates the importance of suspecting brucellosis as a cause of meningoencephalitis even if cultures and serological tests are negative at the beginning of the disease in an endemic area. It was interesting that facial paralysis, vasculitic changes and granulomas developed in our patient during the followup period, mimicking the CNS involvement of tuberculosis, which is also endemic in Turkey. We conclude that brucellosis should not be ruled out in patients who have a history of residence or travel to endemic areas and develop unexplained neurological symptoms.

## Consent

Written informed consent was obtained from the patient's father for publication of this case report and any accompanying images. A copy of the written consent is available for review by the journal's Editor-in-Chief.

## Competing interests

The authors declare that they have no competing interests.

## Authors' contributions

All the authors were involved in the case management. AI performed the literature search and wrote the manuscript. All the authors have read and approved the final manuscript.
